# The Moderating Role of Depression on Momentary Pain-Affect Associations in Osteoarthritis

**DOI:** 10.1093/geroni/igab046.3236

**Published:** 2021-12-17

**Authors:** Emily Behrens, Kyrsten Hill, Dylan Smith, Jason DeCaro, Brian Cox, Patricia Parmelee

**Affiliations:** 1 University of Alabama, Alabama, United States; 2 The University of Alabama, Tuscaloosa, Alabama, United States; 3 Stony Brook University, Stony Brook, New York, United States; 4 University of Alabama, Tuscaloosa, Alabama, United States; 5 University of Alabama, University of Alabama, Alabama, United States

## Abstract

Previous research has found a reciprocal relationship between pain and depression, in which each influences the severity of the other (Chou, 2007; Hawker et al., 2011, Kroenke et al., 2011; Schieir et al., 2009). Studies have found that depressed individuals exhibit stronger pain-mood associations than never-depressed individuals (Conner et al., 2006; Tennen et al., 2006). The current study investigated main and interactive effects of depressive symptoms on the momentary associations between pain and mood. Experience sampling (ESM) data was used from a multi-site study examining individuals with knee osteoarthritis (OA). Participants completed self-report measures of global depression and momentary pain, negative affect (NA), and positive affect (PA). Cross-sectional associations among momentary pain and affect were examined in a series of hierarchical multilevel models that nested the 28 ESM calls (Level 1) within participants (Level 2). A parallel set of multilevel models tested lagged associations among momentary variables. Depression significantly moderated the contemporaneous (p < .001) and lagged (p < .003) associations between pain and NA, suggesting that depression intensifies the momentary pain-NA linkage. There were no significant interaction effects for PA. These findings extend existing knowledge by illustrating how depressive symptoms influence the everyday experience of OA pain and its impact on affective well-being. (Supported by AG041655, P. Parmelee and D. Smith, Co-PIs)

